# *In vitro *activity of pyronaridine against *Plasmodium falciparum *and comparative evaluation of anti-malarial drug susceptibility assays

**DOI:** 10.1186/1475-2875-8-79

**Published:** 2009-04-23

**Authors:** Florian Kurth, Peter Pongratz, Sabine Bélard, Benjamin Mordmüller, Peter G Kremsner, Michael Ramharter

**Affiliations:** 1 Medical Research Unit, Albert Schweitzer Hospital, Lambaréné, Gabon; 2 Institute for Tropical Medicine, Department of Parasitology, University of Tübingen, Tübingen, Germany; 3 Department of Paediatrics, University Hospital Carl Gustav Carus Dresden, Dresden, Germany; 4 Department of Pediatrics and Adolescent Medicine, University Freiburg, Freiburg, Germany; 5 Department of Medicine I, Division of Infectious Diseases and Tropical Medicine, Medical University of Vienna, Vienna, Austria

## Abstract

**Background:**

Pyronaridine, a Mannich base anti-malarial with high efficacy against drug resistant *Plasmodium falciparum*, is currently evaluated as a fixed dose combination with artesunate for the treatment of uncomplicated malaria. In this study, the *in vitro *activity of pyronaridine against clinical isolates of *P. falciparum *from Lambaréné, Gabon, was assessed in order to obtain baseline data on its activity prior to its future use in routine therapy. Moreover, follow-up assessment on the *in vitro *activity of chloroquine, artesunate and quinine was performed.

**Methods:**

*In vitro *response of field isolates of *P. falciparum *to pyronaridine, chloroquine, artesunate and quinine was assessed using the traditional WHO microtest. In addition, the histidine-rich protein 2 (HRP-2) assay was performed and evaluated for its future implementation for follow-up of drug susceptibility testing.

**Results:**

Pyronaridine exhibited a high *in vitro *activity against *P. falciparum*, with a geometric mean cut-off concentration of 9.3 nmol/l. Fifty percent effective concentrations were 1.9 nmol/l and 2.0 nmol/l in the WHO microtest and HRP-2 assay, respectively. Results matched closely *in vivo *findings from a recent clinical trial on pyronaridine-artesunate treatment. One isolate showed diminished sensitivity to artesunate. For chloroquine and quinine resistance levels were comparable to prior studies from Lambaréné. Results from the novel HRP-2 assay corresponded well to those obtained by the WHO microtest.

**Conclusion:**

Pyronaridine is highly active in chloroquine-resistant parasites and seems a promising partner drug for artemisinin-based combination therapy in Africa.

## Background

Malaria continues to be a major cause of morbidity and mortality in sub-Saharan Africa, particularly in young children. Early detection and effective chemotherapy remain the cornerstones in its control [1]. The rapid development and spread of anti-malarial drug resistance has made surveillance of drug sensitivity a high priority issue. In addition to assessing the activity of common anti-malarials against *Plasmodium falciparum *in routine surveys, the evaluation of new compounds against field isolates is of major importance for drug development.

In Lambaréné, Gabon, routine anti-malarial drug susceptibility monitoring has been performed since 1992 [2-5]. So far the WHO microtest – one of the longest used and best validated assays for the assessment of *in vitro *drug sensitivity under field conditions – has been used for this purpose [6]. Meanwhile, novel methods in drug susceptibility testing have been developed, such as the histidine-rich protein II (HRP-2) assay [7]. This method, based on HRP-2 measurement in an enzyme-linked immunosorbent assay (ELISA), is equally simple to implement, but considerably less labour intensive compared with the WHO microtest. Due to these advantages, the WHO microtest will be replaced for standard drug susceptibility monitoring in Lambaréné in the future by the novel HRP-2 assay. Previous findings from laboratory adapted clones and from different geographical regions suggest that results obtained by the HRP-2 assay be comparable with those obtained by the WHO microtest [7].

The anti-malarial agent pyronaridine is a Mannich base derivative of mepacrine, one of the earliest synthetic anti-malarials [8]. It is currently evaluated as a fixed dose combination with artesunate for the treatment of uncomplicated falciparum and vivax malaria in adult and paediatric patients [9]. Its anti-plasmodial activity involves interference with the glutathione-dependent detoxification of haem and targeting of β-haematin formation [10]. Reports from paediatric patients in Africa showed that pyronaridine is effective against chloroquine resistant strains of *P. falciparum in vivo *[9,11], yet data from South-East Asia indicate the potential for rapid development of resistance against pyronaridine, when used as monotherapy [12].

The present study aimed to assess the susceptibility of clinical *P. falciparum *field isolates from Lambaréné to pyronaridine in order to obtain baseline data on the activity of this for Africa yet novel anti-malarial drug prior to its widespread use in routine therapy. In addition the study was designed to assess the potential for the novel HRP-2 assay to replace the standard WHO microtest.

## Methods

### Study area and patients

The study was carried out at the Medical Research Unit of the Albert Schweitzer Hospital in Lambaréné, Gabon, in a region of stable, hyperendemic *P. falciparum *malaria transmission [13,14]. Patients attending the outpatient clinic between March and October 2006 were asked to participate in the study if they met the following inclusion criteria: *P. falciparum *monoinfection with 1,000–100,000 asexual parasites per μl blood, no schizontaemia, no signs or symptoms of severe malaria, and no history of intake of anti-malarial drugs in the preceding month. Information about age, sex, and duration of fever was collected on a questionnaire. Informed consent was obtained from participants or their legal representatives. The study was approved by the Ethics Committee of the International Foundation for the Albert Schweitzer Hospital in Lambaréné.

### *In vitro *drug sensitivity assays

Two different methods of drug sensitivity testing were deployed in order to assess the susceptibility of fresh field isolates of *P. falciparum *to pyronaridine, artesunate, chloroquine and quinine. First, the standard World Health Organization *in vitro *microtest was used similar to previous reports, measuring drug-dependent inhibition of schizont maturation (SMI) within 24 hours by microscopic assessment [2-5]. Briefly, two millilitres of venous blood were mixed with complete parasite culture medium (RPMI 1640, 200 μM hypoxanthine, 25 mM Hepes, 0.5% albumax, 2 mM l-glutamine) to a final concentration of 5% blood medium mixture (BMM). Ninety-six-well test plates were pre-dosed in ascending quantities of drugs, dosing each plate with all respective drugs for one isolate. Final drug concentrations were 0.5–365.9 nmol/l BMM for pyronaridine (Mr: 910.04), 0.1–85.8 nmol/l BMM for artesunate (Mr: 384.425), 0.8–51.2 μmol/l blood for chloroquine (Mr: 515.867) and 55–3567 nmol/l BMM for quinine (Mr: 785.06). Artesunate, chloroquine and quinine were dissolved in 70% ethanol, pyronaridine was resuspended in distilled water. In accordance to the protocols distributed by the World Health Organization drug concentrations for chloroquine are expressed as related to blood due to their considerable accumulation in erythrocytes [6].

Fifty μl BMM were transferred into scheduled wells and incubated at 37.5°C in candle jars. After 24 hours parasites were harvested and Giemsa-stained thick blood films were prepared. The number of mature schizonts was microscopically counted against 200 asexual parasites in each well. Tests were considered successful if at least 10% schizont maturation was observed in the drug-free control-well.

In addition, the HRP-2 assay was performed according to the published standard operational procedures [7]. Two ml of venous blood were mixed with parasite medium to a concentration of 3% BMM. Test plates were pre-dosed to the same final concentrations as in the WHO test and incubated for 72 hours at 37°C in candle jars. To test for successful *in vitro *parasite-growth, a thick blood smear of one control-well was performed after 26 h. One non-treated 26 h sample was frozen to calculate background HRP-2 production. Parasite culture was judged successful if at least 10% parasites matured to schizonts at the 26-hour time point. After 72 h plates were freeze-thawed twice. Parasite growth, calculated from HRP-2 levels, was measured with an enzyme linked immunosorbent assay at an absorbent maximum of 450 nm.

### Statistical analysis

Non-linear regression analysis with 4-parameter fits of log-concentration/response curves was used to determine individual inhibitory concentrations of the respective isolates. All regressions were checked manually. Cut off concentrations were calculated as geometric mean of the lowest individual concentrations with no mature schizont among 200 parasites in the WHO microtest. Nonparametric analysis was used for concentration data that was not normally distributed. A two-tailed Mann-Whitney-U-Test was performed in order to test for difference between the two drug sensitivity assays. All tests were performed at a two sided significance level of α = 0.05.

## Results

Ninety-five outpatients attending the Albert Schweitzer Hospital were included in this study. The patients' median age was three years, ranging from three month to 18 years, and 55% were female. Median asexual parasitaemia at presentation was 36,500 per μl blood. In the schizont maturation microtest 32, 36, 34, and 32 out of 95 isolates yielded valid results for pyronaridine, chloroquine, artesunate and quinine, respectively. Twenty-five isolates fulfilled the criteria for successful parasite-culture in the HRP-2 assay and 15, 16, 12 and 14 were successfully employed for drug sensitivity testing, respectively. Post-hoc analysis of the unexpected low success rate of cultivation, especially in the HRP-2 assay, revealed that poor growth was associated with one lot of the commercially acquired medium. Those isolates were identified and excluded from the analysis. Table 1 shows 50 percent, 99 percent and cut-off concentrations of the tested drugs as obtained by the WHO microtest and HRP-2 assay.

**Table 1:** EC_50_, EC_99 _and cut-off concentrations of pyronaridine, chloroquine, artesunate and quinine in WHO microtest and HRP-2 assay

	WHO Microtest			HRP-2 assay		
	*N*	EC_50 _(95%CI)	Cut-off concentration (95%CI)	*N*	EC_50 _(95% CI)	EC_99 _(95%CI)
Pyronaridine	32	1.87 (1.40–2.48)	9.32 (5.2–13.9)	15	2.03 (1.57–2.63)	9.03 (6.42–12.69)
Chloroquine	36	7.1 (5.2–9.6)	21.7 (15.4–30.6)	16	5.5 (4.4–6.9)	19.2 (15.3–24.2)
Artesunate	34	2.08 (1.48–2.92)	9.24 (6.53–13.06)	12	2.25 (1.70–2.99)	15.24 (10.45–22.24)
Quinine	32	272 (210–353)	873 (723–1053)	14	204 (167–250)	662 (561–781)

All values are in nmol/l BMM except for chloroquine (μmol/l blood),

Means and 95% confidence intervals are depicted as antilog of arithmetic mean of log-transformed data

### Pyronaridine

Pyronaridine inhibited *in vitro *growth and schizont maturation in the WHO assay at a geometric mean cut-off concentration of 9.3 nmol/l BMM. The respective geometric mean EC_99 _concentration in the HRP-2 assay was 9.0 nmol/l. In one isolate schizont maturation was observed at a pyronaridine concentration of 122 nmol/l BMM, resulting in a cut-off concentration of 366 nmol/l BMM. Further two isolates showed cut-off concentrations of 122 nmol/l. Geometric mean EC_50 _values were 1.9 and 2.0 nmol/l BMM for the WHO microtest and the HRP-2 assay, respectively.

### Chloroquine

The geometric mean cut-off concentration for chloroquine was 21.7 μmol/l blood in the WHO assay, 19.2 μmol/l blood was the respective EC_99 _value in the HRP-2 assay. The most sensitive isolate showed a cut-off concentration of 3.2 μmol/l blood, all other isolated exhibited cut-off concentrations of 6.4 μmol/l blood or higher. In four parasite cultures, microscopic assessment revealed schizont maturation up to the highest concentration of 51.3 μmol/l blood. The 50 percent effective concentrations were 7.1 μmol/l blood in the WHO microtest and 5.5 μmol/l in the HRP-2 assay, respectively.

### Artesunate

Fifty percent effective concentrations were 2.1 nmol/l BMM in the microtest and 2.3 nmol/l BMM in the HRP-2 assay for artesunate. Two isolates exhibited schizont maturation at 28.6 nmol/l BMM, yielding cut-off concentrations of 85 nmol/l BMM. In one isolate *in vitro *schizont maturation was not inhibited by the highest artesunate concentration of 85 nmol/l BMM. The same isolate exhibited good sensitivity to pyronaridine and to quinine. The geometric mean cut-off concentration was 9.2 nmol/l BMM. The HRP-2 assay showed a mean EC_99 _level of 15.2 nmol/l BMM.

### Quinine

All isolates were susceptible to quinine. Highest cut-off concentrations were at 1783 nmol/l BMM, thus well below the threshold of resistance (5120 nmol/l BMM). The geometric mean cut-off concentration was 873 nmol/l, the EC_99 _in the HRP-2 assay was 662 nmol/l BMM. The EC_50 _values in the WHO microtest and in the HRP-2 assay were 272 and 204 nmol/l BMM, respectively.

### Activity correlation of the tested drugs

Correlation analysis of EC_50 _values of the individual isolates was performed in order to assess the relationship between sensitivity of parasites to pyronaridine, chloroquine, artesunate and quinine (Table 2). Significant correlation was found between *in vitro *activities of the anti-malarials quinine and chloroquine (r = 0.50, p < 0.005). Interestingly, the activity of pyronaridine was correlated with artesunate (r = 0.84 p < 0.0001), but not with chloroquine or quinine.

**Table 2:** Activity correlation between pyronaridine, chloroquine, artesunate and quinine in fresh *Plasmodium falciparum *isolates

		**Pyronaridine**	**Chloroquine**	**Artesunate**
**Chloroquine**	R^2^	**0.13**	-	-
	P	0.52		
	N	30		
**Artesunate**	R^2^	**0.84**	-**0.07**	-
	P	<.0001*	0.69	
	N	29	30	
**Quinine**	R^2^	**0.06**	**0.50**	**0.02**
	P	0.74	0.0037*	0.93
	N	30	31	30

R^2^: correlation coefficient in pairwise correlation analysis

* statistically significant

### Comparison of HRP-2 assay and WHO microtest

**Figure 1:**
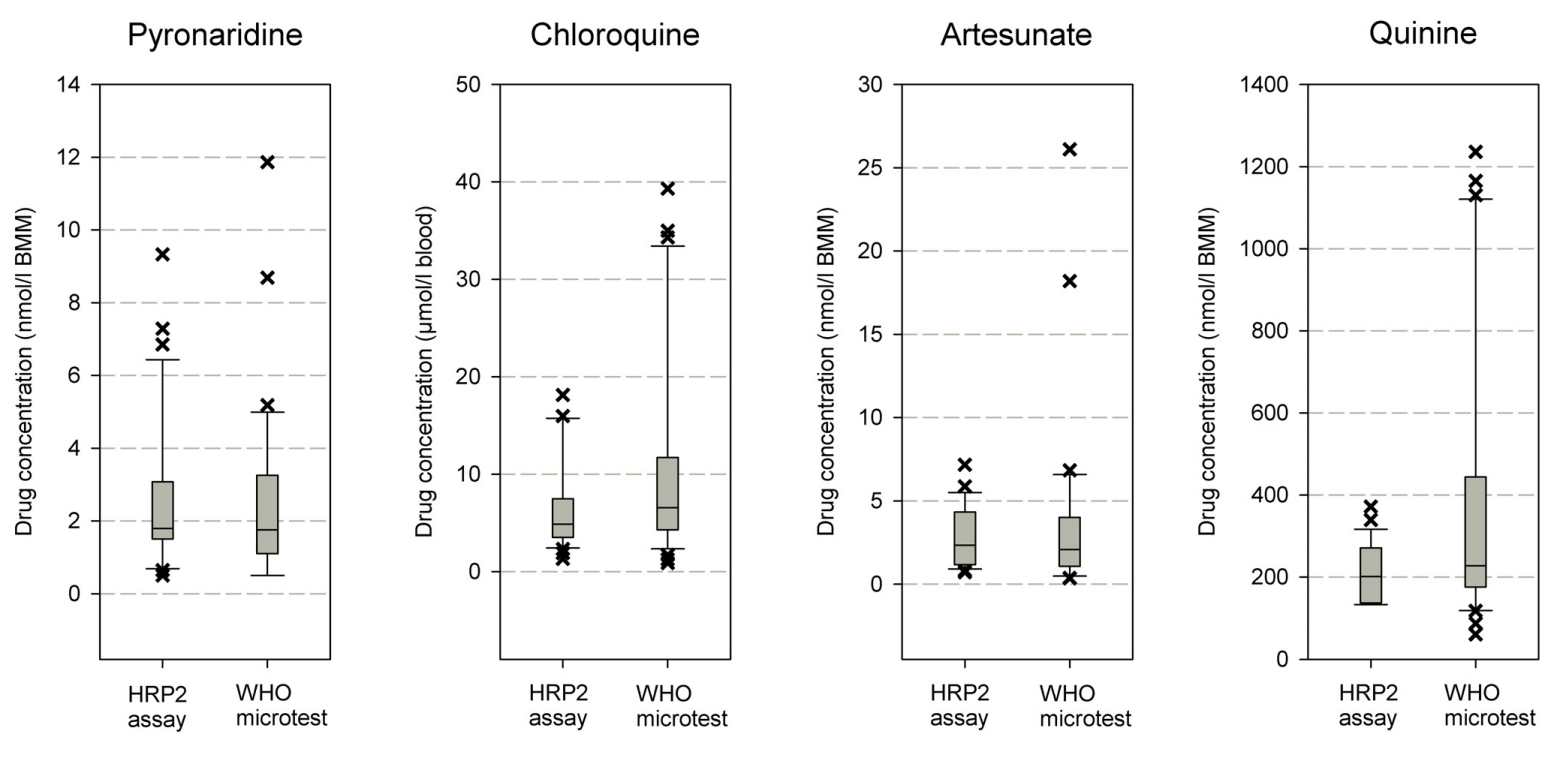
**Box plots of pyronaridine, chloroquine, artesunate and quinine EC_50 _in HRP-2 assay and WHO microtest**.

No statistically significant differences between the EC_50 _values from the two drug sensitivity assays were found in U-test of pooled data of the individual drugs (p = 0.82 for pyronaridine, 0.72 for artesunate, 0.11 for chloroquine and 0.20 for quinine). Differences in geometric means of EC_50 _values were largest for chloroquine (7.1 μmol/l, 95% CI 5.2–9.6 in the WHO microtest versus 5.5 μmol/l 95% CI: 4.4–6.9 in the HRP-2 assay, p = 0.11) and quinine (272 nmol/l 95% CI: 210–353 versus 204 nmol/l 95%CI 167–250, respectively. p = 0.20). Figure 1 shows the distribution of EC_50 _values for the respective drugs.

Cut-off concentrations as determined by the WHO microtest correlate closely to the equivalent EC_99 _values of the HRP-2 assay (Table 1). Significantly more isolates yielded valid results in the microtest (36%) than in the HRP-2 based assay (15%) (p < 0.001).

## Discussion

Pyronaridine exhibited a high level of *in vitro *activity against isolates of *P. falciparum *from infected Gabonese subjects in this study. Fifty percent effective concentrations were 2.0 and 1.9 nmol/l, which is consistent with previously published data from studies with laboratory strains and field isolates obtained by a different drug sensitivity assay (isotopic hypoxanthine assay) [15,16].

Median pyronaridine concentrations of 9.3 nmol/l (EC_99 _in HRP-2 assay) and 9.0 nmol/l (cut-off concentration in WHO microtest) inhibited growth of *P. falciparum in vitro*. In a recent pharmacokinetic assessment of a fixed-dose pyronaridine artesunate combination in African children, the mean maximal plasma concentrations of pyronaridine were 115 nmol/l in patients [9]. Earliest reappearances of parasites in peripheral blood in this study were observed on day 21 (one patient) and day 28 (three patients) after treatment. Interestingly, the patient with parasite reappearance on day 21 exhibited a pyronaridine plasma level of 9.9 nmol/l at that time. In polynomial regression analysis of pooled plasma levels of all patients, mean pyronaridine blood concentrations fell below levels of 9 nmol/l on day 22. First reappearances *in vivo *therefore occurred only after plasma levels fell below concentrations similar to cut-off concentrations obtained in this *in vitro *study.

Although the extrapolation of *in vitro *drug sensitivity assessments to *in vivo *data should always be cautious [17], *in vitro *results of the present study matched closely clinical findings of this clinical trial on pyronaridine-artesunate combination therapy.

The relationship between *in vitro *activity of pyronaridine and chloroquine against *P. falciparum *has been discussed controversially in previous reports and contradictory conclusions concerning cross-resistance have been drawn from studies, which found a significant correlation between pyronaridine and chloroquine *in vitro *activity and differences in activity of pyronaridine against chloroquine resistant and sensitive strains [15,16,18,19]. In the present study, *in vitro *response to chloroquine was poor in all tested isolates, yet parasites proved to be highly susceptible to pyronaridine. There was no statistically significant correlation of the *in vitro *activities of the two drugs against *P. falciparum*. These *in vitro *findings and the recently reported high cure rates of three days pyronaridine-artesunate combination therapy provide convincing evidence against a clinically significant cross resistance of pyronaridine and chloroquine *in vivo*. The results underline the usefulness of pyronaridine for the treatment of patients in regions affected by *P. falciparum *resistant to chloroquine [9,11].

Since the first *in vitro *drug susceptibility assessment in Lambaréné in 1992 [2], high levels of resistance against chloroquine have been observed *in vitro *and *in vivo *[3-5]. Similar to previous studies *in vitro *response to chloroquine was poor in this study. EC_50 _levels were comparable to the last assessment in 2002 [5], despite a decrease in drug pressure due to a change of national treatment policies to artemisinin-based combination therapy in 2003. Artesunate was highly active against *P. falciparum *in the present assessment. EC_50 _levels were similar to recent *in vitro *and *in vivo *findings at the study site and in neighbouring countries [20-22]. One isolate showed a diminished sensitivity to artesunate with schizont maturation up to the highest concentration of 85.8 nmol/l and a corresponding EC_50 _level of 19.9 nmol/l. Another isolate had a comparably high EC_50 _value of 10.1 nmol/l and a respective cut-off concentration in the WHO microtest of 85.8 nmol/l. These findings support recent *in vitro *susceptibility assessments by Cojean *et al *[23], reporting on 6 out of 397 African *P*. *falciparum *isolates with dihydroartemisinin EC_50 _levels above 10 nmol/l and a maximum EC_50 _of 31.8 nmol/l in one isolate. Especially in the light of increasing availability and use of artemisinins in many parts of Africa, the importance of close surveillance for susceptibility as well as strict deployment of artemisinins exclusively in combination therapy with effective partner drugs cannot be overemphasized.

As in previous reports from Lambaréné, susceptibility to quinine was high in this study. Mean EC_50 _(204 and 272 nmol/l in HRP-2 assay and WHO microtest, respectively) were comparable to results obtained in 2002 (286 nmol/l) [5]. Despite its widespread use, quinine remains a highly effective anti-malarial in Gabon, especially as drug of choice for parenteral treatment of hospitalized patients.

Comparative assessment of the HRP-2 assay and WHO microtest was performed in order to establish baseline data for the use of the ELISA based assay in Lambaréné in the future. Results obtained by the HRP-2 assay closely paralleled those obtained by the WHO microtest despite methodological problems in this study due to a deficient batch of culture medium. Considering its reduced labour-intensity and good reproducibility the HRP-2 assay can, therefore, be recommended for follow up of anti-malarial drug susceptibility testing and will replace the WHO microtest in future assessments at our study site.

## Conclusion

Artesunate, chloroquine and quinine *in vitro *drug activity was similar, compared to data from earlier *in vitro *assessments, and results of the HRP-2 assay were comparable to those obtained by the traditional WHO microtest.

The study demonstrated high anti-malarial activity of pyronaridine against fresh field isolates of *P. falciparum *and corresponded well to recent findings of pyronaridine anti-malarial activity *in vivo*. Pyronaridine is recommended for further clinical development in combination therapy and continued *in vitro *drug activity monitoring.

## Competing interests

The authors declare that they have no competing interests.

## Authors' contributions

FK contributed to the conception and design of the study, performed parasite cultivation and microscopic assessment, analysed the data and wrote the manuscript. PP performed parasite cultivation and HRP-2 immunoassay. SB gathered field isolates from patients and performed parasite cultivation. BM contributed to performance of immunoassay and analysis of data, PGK revised the manuscript and supervised the research group, MR conceived and designed the study, contributed to analysis of data, performed second reading for microscopic assessments in WHO microtest and drafted and revised the manuscript. All authors read and approved the final version of the manuscript.
